# Seawater-Associated Highly Pathogenic *Francisella hispaniensis* Infections Causing Multiple Organ Failure

**DOI:** 10.3201/eid2610.190844

**Published:** 2020-10

**Authors:** Hua Zhou, Qing Yang, Lisha Shen, Yake Yao, Jun Xu, Junhui Ye, Xiaomai Wu, Yunsong Yu, Ziqin Li, Jianying Zhou, Shangxin Yang

**Affiliations:** The First Affiliated Hospital of Zhejiang University School of Medicine, Hangzhou, China (H. Zhou, L. Shen, Y. Yao, J. Xu, J. Zhou);; State Key Laboratory for Diagnostic and Treatment of Infectious Diseases, The First Affiliated Hospital of Zhejiang University School of Medicine, Hangzhou (Q. Yang);; Sanmen People’s Hosptial, Taizhou, China (J. Ye);; Taizhou Hospital, Taizhou (X. Wu);; Sir Run Run Shaw Hospital of Zhejiang University School of Medicine, Hangzhou (Y. Yu);; Zhejiang-California International Nanosystems Institute, Zhejiang University, Hangzhou (Z. Li, S. Yang);; UCLA School of Medicine, Los Angeles, California, USA (S. Yang)

**Keywords:** Francisella hispaniensis, seawater, multiorgan failure, bacteria, wound, bacteremia, moxifloxacin, China

## Abstract

A rare case of *Francisella hispaniensis* infection associated with seawater exposure occurred in a deep-sea diving fisherman in Zhejiang, China. He had skin and soft tissue infection that progressed to bacteremia and multiple organ failure. Moxifloxacin treatment cleared the infections, but the patient suffered a sequela of heart damage.

*Francisella tularensis*, the agent of tularemia, is an important human pathogen (*1*). Other *Francisella* species, such as *F. philomiragia*, mainly associated with saltwater exposure, rarely also cause human infections (*2*). *F. hispaniensis*, first isolated from the blood of a patient in Spain (*3*), is an emerging human pathogen, but its epidemiology and pathogenicity remain a mystery because only 2 cases have been reported (*3*,*4*). We report a case of *F. hispaniensis* infection in China.

## Case Report

**Figure 1:**
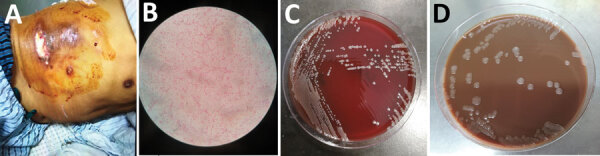
Chest wound of a 64-year-old male fisherman and isolated bacteria morphology, China. A) Ulcer and necrosis in the lower chest. B) Gram-negative cocci isolated from blood and wound. C) Growth on blood agar after 5 days with CO_2_. D) Growth on chocolate agar after 5 days with CO_2_.

On September 6, 2018, a 64-year-old male fisherman sought care for a prominent cutaneous ulcer on the right lower chest, chest pain, and fever for 16 days and was admitted to The First Affiliated Hospital of Zhejiang University (Hangzhou, China). He was previously healthy without remarkable medical history. He worked as a deep-sea diving fisherman in Sanmen Bay, Taizhou, a coastal city in Zhejiang Province, adjoining the East China Sea. A superficial wound progressed to cellulitis in the right lower chest after a deep-sea dive without protective clothing. Low-grade fever and chest pain then developed. He received amoxicillin/clavulanic acid at a local clinic, and his fever resolved after 2 days. Because he felt better, he stopped taking the amoxicillin/clavulanic acid and resumed deep-sea diving. Two days later, his chest wound had worsened with purulent discharge, and his low-grade fever returned. Twelve days later he sought care at another hospital because of high fever and respiratory distress. He received 2 days of ceftizoxime followed by imipenem for 7 days, but his condition deteriorated, and irritability, chest tightness, nausea, vomiting, abdominal distension, chills, and high fever (39.4°C) developed. At admission to The First Affiliated Hospital of Zhejiang University School of Medicine, he had sepsis, hypotension, and leukocytosis and immediately received norepinephrine intravenous pumping, endotracheal intubation, sedation, mechanical ventilation, and continuous renal replacement therapy. His lower chest showed a large ulcer with bleeding, purulent discharge, and tissue necrosis (Figure 1, panel A). Laboratory test results showed highly elevated inflammatory markers, acidosis, coagulopathy, and elevated liver enzymes, bilirubin, creatinine, and troponin (Table 1). Chest computed tomography scan showed right lower lobe consolidation, pleural effusion in the right thoracic cavity, and multiple calcified lymph nodes in the mediastinum. Abdomen computed tomography scan showed hepatosplenomegaly and effusion in the abdominal and pelvic cavities. Echocardiography showed decreased left ventricular systolic function and diffuse abnormal movement of left ventricular wall. Electrocardiograph showed cardiac arrhythmia with sinus bradycardia, ventricular premature beats, and paroxysmal ventricular tachycardia. Acute diffuse myocarditis was diagnosed and prompted dobutamine treatment.

**Table 1:** Blood test results during progression of *Francisella hispaniensis* infection and after treatment of a 64-year-old fisherman with multiple organ failure, China

Blood test (reference range)	Outside hospital, 4 d after fever onset	At admission, 16 d after fever onset	After treatment, 14 d after admission
Leukocytes, cells/mm^3^ (4,000–10,000)	16,600	22,800	10,700
Differential count, %			
Neutrophils (50–70)	90.6	81.1	84.2
Lymphocytes (20–40)	5.1	16	8.7
Platelets/mm^3^ (83,000–303,000)	174,000	145,000	159,000
Hemoglobin, g/dL (13.1–17.2)	13.4	10.7	6.8
Creatinine, mg/dL (0.7–1.2)	0.6	2.6	1.5
Albumin, g/dL (3.5–5.5)	2.97	2.81	2.72
Alanine aminotransferase, U/L (5–40)	47	394	27
Aspartate aminotransferase, U/L (8–40)	53	1911	22
Total bilirubin, mg/dL (0–1.3)	0.9	6.3	1.2
Direct bilirubin, mg/dL (0–0.3)	0.4	4.8	0.8
Activated partial thromboplastin time, s (14.5–21.5)	40.1	82.5	36.5
Prothrombin time, s (10.0–13.5)	15.1	40.8	12.8
Fibrinogen, g/L (2.0–4.0)	8.83	1.28	3.2
Troponin I, ng/mL (0–0.06)	Not available	1.13	0.1
N-terminal pro-brain natriuretic peptide, pg/mL (0–80)	Not available	>9,000	1,845
Arterial blood pH (7.35–7.45)	7.45	7.21	7.48
Arterial partial pressure of oxygen, mm Hg (80–100)	50	130	138
Arterial partial pressure of carbon dioxide, mm Hg (35–45)	32	36	32
Lactate, mmol/L (0.5–2.2)	Not available	14.2	1.6
C-reactive protein, mg/L (0–8)	Not available	292.6	96.8
Procalcitonin, ng/mL (0–0.5)	Not available	12.84	0.30

**Figure 2:**
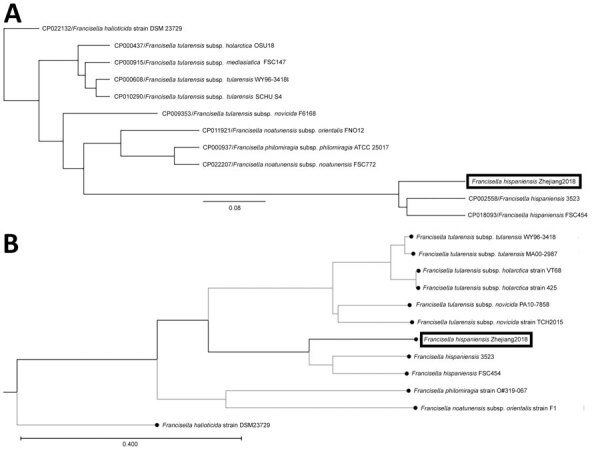
Comparisons of *Francisella* *hispaniensis* isolate from a 64-year-old male fisherman, China (black boxes), and reference sequences. A) Single-nucleotide polymorphisms. Scale bar for indicates expected substitutions per nucleotide position. B) k-mer phylogenetic tree. Scale bar indicates the branch lengths within the tree.

Blood, pleural fluid, and wound culture all grew gram-negative cocci (Figure 1, panel B), identified by Vitek2 (bioMérieux, https://www.biomerieux.com) as *Sphingomonas paucimobilis*. The bacteria grew well on the regular sheep blood agar and showed medium-sized, smooth-edged, mucoid and greyish white colonies (Figure 1, panel C). They grew better on chocolate agar (Figure 1, panel D) but did not grow on MacConkey agar. The bacteria were catalase weakly positive, oxidase positive, indole negative, and β-lactamase positive. Because *S. paucimobilis* is usually considered an environmental bacterium and unlikely to cause such severe systemic infections, we sent the patient’s blood for shotgun metagenomic sequencing test and the bacterial isolate for whole-genome sequencing (WGS) using Illumina MiniSeq (https://www.illumina.com). Metagenomic sequencing yielded a positive result as *F. tularensis*, but WGS identified *F. hispaniensis*, on the basis of k-mer and single-nucleotide polymorphism phylogenetic tree analyses performed using CLCbio (QIAGEN, https://www.qiagen.com) (Figure 2), which showed the bacteria clustered closely with 2 other *F. hispaniensis* strains (*3*,*4*) and very distantly with other *Francisella* species. To verify the results, we mapped the raw sequencing reads to the most closely related reference genome *F. hispaniensis* FSC454 (GenBank accession no. CP018093) using Geneious (BioMatters, https://www.geneious.com), which resulted in 96.1% genome coverage with 97.9% pairwise identity. The FSC454 and Zhejiang2018 strains differ by only 1 nt (A1029G) in the 16S rRNA gene (99.94% identity) and 10 nt changes in the *recA* gene (99.07% identity).

Drug susceptibility tests showed resistance to colistin, trimethoprim/sulfamethoxazole, third-generation cephalosporins, and carbapenems but susceptibility to piperacillin/tazobactam, cefepime, fluoroquinolones, aminoglycosides, and tetracyclines (Table 2). Because a Bla-2/FTU-1 class-A β-lactamase is expressed among most *Francisella* species (*6*), the strain reported here also carries a homologue gene of 867 bp with 89.7% identity to the reference gene (GenBank accession no. NG_049110_FTU-1) (*7*). No plasmids were identified. Other resistance genes identified were *aph(3¢)-Ia*, predicting resistance to kanamycin; *mdf(A)*, predicting resistance to macrolide; and *catA1*, predicting resistance to phenicol. However, broth microdilution tests showed low MIC for kanamycin, erythromycin, azithromycin, and chloramphenicol (Table 2). The reason for the inconsistency between the resistance genes detected and phenotypic susceptibility results is unclear and requires further investigation.

**Table 2:** Drug susceptibility testing results of a *Francisella hispaniensis* isolate from a 64-year-old fisherman, China

Antimicrobial drug	Interpretation*	MIC, μg/mL
Amikacin	S	<2
Colistin	R	>16
Levofloxacin	S	≤0.12
Trimethoprim/sulfamethoxazole	R	>320
Tobramycin	S	<1
Piperacillin/tazobactam	S	<4
Cefoperazone/sulbactam	R	>64
Ciprofloxacin	S	<0.25
Imipenem	R	>16
Minocycline	S	<1
Ceftazidime	R	>64
Cefepime	S	4
Meropenem	R	>16
Tigecycline	S	<0.5
Kanamycin	NA	2
Chloramphenicol	NA	2
Erythromycin	NA	1
Azithromycin	NA	0.5
Amoxicillin/clavulanic acid	NA	>32

*Interpretation was based on the breakpoints for Non-*Enterobacteriaceae* (*5*). NA, breakpoint not available; R, resistant; S, susceptible.

On the basis of the MIC results and the literature (*4*,*8*,*9*), we chose moxifloxacin (400 mg 1×/d injection) to treat the infection. After 14 days of treatment, the patient’s symptoms markedly improved, and the chest wound started to heal. Most blood test results had returned to normal ranges (Table 1). However, his heart suffered long-term damage because of the myocarditis, and he required a pacemaker. He was discharged 28 days after admission.

## Conclusions

In the 2 previously reported human *F. hispaniensis* infections, the bacteria were isolated from blood (*3*,*4*). The patient we report first suffered a trauma to unprotected skin of his chest that was exposed to seawater from which the bacteria entered the wound and caused the skin and soft tissue infections that progressed to bacteremia and sepsis. Like *F. tularensis*, *F. hispaniensis* appeared to be highly pathogenic and caused respiratory failure, septic shock, and multiple organ dysfunction syndrome. Unlike *F. tularensis*, *F. hispaniensis* grew well under regular culture condition. However, because of its rarity in the clinical setting, conventional biochemical methods misidentified the bacterium as *S. paucimobilis*. WGS is a powerful molecular method to provide the definitive identification.

Most interestingly, the bacteria appeared to have originated from seawater. Sanmen Bay has muddy beaches with shallow seawater and high microbial richness suitable for marine aquaculture (http://www.sanmen.gov.cn/art/2018/6/5/art_1519452_20483713.html). *F. hispaniensis* also probably lives in seawater and under the right conditions could cause human infections. In 1 *F. hispaniensis* case, a woman in Australia had a fishhook injury, which was consistent with the seawater exposure hypothesis (*4*). Other *Fransicella* species, such as *F. noatunensis*, which inhabits the ocean, are major pathogens for fish and shellfish (*10*). The patient in our report acquired infection in August, the hottest month in Zhejiang Province. The high temperature could promote bacteria growth in the seawater and increase the likelihood of human exposure.

The *F. hispaniensis* isolate in our report exhibited a similar antimicrobial susceptibility pattern to *F. tularensis*. This finding is consistent with a study showing susceptibility of all 91 *Francisella* strains tested to aminoglycosides, tetracycline, and fluoroquinolones (*11*). Fluroquinolones, such as ciprofloxacin, are highly effective in treating infections caused by *F. tularensis* (*12*), *F. philomiragia* (*2*,*13*), *F. novicida* (*14*), and *F. hispanensis* (*4*). Third-generation cephalosporins and carbapenems are generally not active against *Francisella* spp. (*9*,*11*), as shown by failed treatment with ceftizoxime and imipenem in the case we describe. Studies based on mouse models showed moxifloxacin is more effective than ciprofloxacin in treating tularemia and is less affected by treatment delay (*9*,*15*). In this patient, moxifloxacin successfully treated *F. hispaniensis* infections without relapse.

In summary, clinicians need to be aware of the emerging and highly pathogenic *F. hispaniensis*, which is resistant to many β-lactams, including the cephalosporins and carbarpenems commonly used for empirical treatment. Our report also demonstrates that seawater exposure can be a risk factor for acquiring *F. hispaniensis* infection.
